# Revisiting mental accounting classic paradigms: Replication Registered Report of the problems reviewed in Thaler (1999)

**DOI:** 10.1098/rsos.250979

**Published:** 2025-09-17

**Authors:** Mengfei Li, Gilad Feldman

**Affiliations:** ^1^ Department of Psychology, The University of Hong Kong, Hong Kong

**Keywords:** mental accounting, bias, judgement and decision-making, behavioural economics, registered report, replication

## Abstract

Mental accounting, the internal categorization system individuals adopt to manage their financial activities, may result in suboptimal decisions or decision-making not aligned with one’s own goals. In a Registered Report with an online US sample recruited from Amazon Mechanical Turk using CloudResearch, we conducted a replication of 17 classic problems reviewed in Thaler 1999 *J. Behav. Decis. Mak.* **12**, 183–206. (doi:10.1002/(SICI)1099-0771(199909)12:33.0.CO;2-F) (*N* = approx. 500 per problem; overall: *N* = 1007). We concluded a mostly successful replication: out of the 17 problems, we found empirical support for 11, mixed empirical support for three and no empirical support for three. Extending the replication, we provided an initial test of four untested predictions described in Thaler 1999 *J. Behav. Decis. Mak.* **12**, 183–206. (doi:10.1002/(SICI)1099-0771(199909)12:33.0.CO;2-F), of which we found empirical support for two, mixed support for one and no support for one. Materials, data, and analysis code are available on: https://osf.io/v7fbj/. This Registered Report has been officially endorsed by Peer Community in Registered Reports: https://doi.org/10.24072/pci.rr.100375.

## 1. Background

Mental accounting is an internal control system that individuals use to evaluate, manage and monitor their financial activities [1]. By utilizing this set of cognitive operations, people aim to simplify their financial decision-making process, yet mental accounting may lead to decisions that seem to violate fundamental neoclassical economic principles. To summarize this literature, Thaler [1] wrote a seminal review summarizing over a decade of observations and empirical findings that relate to mental accounting.

Thaler [1] focused on the three most noticeable components of the mental accounting paradigms. First, Thaler [1] pointed out that mental accounting describes how people perceive and experience outcomes. It explains how people make and evaluate their financial decisions. Second, grouping expenses into categories is another defining feature of mental accounting [2]. The mental accounting system demonstrates how different activities are assigned into specific separate accounts. For example, Heath and Soll [3] suggested that expenses must first be ‘booked’ and then ‘posted’ into proper accounts with reference to the similarity and categorization. Third, mental accounting concerns how choices are grouped together and how frequently people evaluate the mental accounts. Individuals and households can balance accounts on a daily, monthly or a yearly basis and can define the accounts either narrowly or broadly [1]. Mental accounting is comparable with financial accounting that businesses conduct to monitor expenditures [4].

We report a very close replication of the studies reviewed in Thaler [1] (replication closeness evaluation based on LeBel *et al.*’s [5] criteria). Our first goal was to conduct independent close well-powered replications of the classic effects reviewed by Thaler [1]. Our second goal was to empirically test several predictions made in Thaler [1] that the review did not provide empirical tests for.

We begin by introducing the literature on mental accounting and the chosen review article for replication—Thaler [1]. We then detail our motivations for the current replication study and provide an overview of the problems covered in our replication.

### 1.1. Mental accounting

Mental accounting has long been a heated topic in the field of behavioural economics, psychology, and judgement and decision-making. The earliest empirical evidence on mental accounting behaviours dates back to Tversky and Kahneman’s [6] famous theatre-ticket experiment (one of our replication problems). In that study, participants were asked whether they would be willing to pay $10 for a ticket following a loss, and the authors contrasted two conditions that manipulated whether the participants had lost a previously purchased ticket for the same show or lost an equivalent $10 bill. The results showed that people were less willing to purchase the ticket after losing a ticket compared with after losing an equivalent cash amount.

Tversky and Kahneman proposed that mental accounting is a form of decision framing by which people formulate (psychological) accounts to evaluate events and options, as cited in [7]. People categorize funds into different mental accounts designed for different purposes. Participants probably perceive the funds required to ‘repurchase’ the ticket as drawn from the mental account for ticket expenditures, which had already been used in the initial purchase. In contrast, the cash loss was not assigned to a discrete mental account. This distinction violates the long-standing economic notion of fungibility [1].

Ever since, the concept of mental accounting has been used to understand a wide range of decision-making behaviours, such as gambling, risk-taking and investment [8]. Although these subsequent mental accounting studies differ in specific objectives and orientations, nearly all research has touched upon gains and losses and indicated the violation of fundamental economic norms [7]. In Thaler [9] and in Thaler [1], our target article, the mental accounting phenomenon was further elaborated and expanded into a broader theory of decision-making and choice [10]. In 2011, Soman and Ahn [11] reviewed substantial mental accounting research focusing on framing effects. More recently, Zhang and Sussman’s [2] review paper again outlined the categorization process of mental accounting, and they summarized it as a way for people to ‘group expenses into categories, assign funds to these categories, determine budgets and perform elements of cost–benefit analyses’. (p. 65). A very similar set of papers (e.g. [3,6,8,12]) served as the basis for Thaler [1] and the two recent review papers [2,11]. This further exemplifies the necessity of revisiting these classic findings and testing the reproducibility, robustness and generalizability of these influential and pioneering works, to substantiate and strengthen the empirical foundations of the theoretical framework of mental accounting. We therefore aimed to revisit the evidence reviewed in Thaler [1] and re-examine the different subsets of the mental accounting framework.

### 1.2. Choice of article for replication: Thaler, 1999

We chose the Thaler [1] article based on three factors: its extensive academic impact, the need for systematic direct replications of many studies covering a single domain, and the potential in methodological improvements in classic older studies.

As of May 2025, the time of writing, there were 5912 Google Scholar citations of the review article and much important follow-up theoretical and empirical research. The review has had an immense impact on scholarly research in the area of behavioural economics, judgement and decision-making, and consumer psychology, with the research covered in Thaler [1] becoming highly influential. We summarized the citations impact of each of the problems covered in the article in table 1. Thaler received the Sveriges Riksbank Prize in Economic Sciences in Memory of Alfred Nobel in the year 2017, recognizing mental accounting among Thaler’s most influential work—‘Thaler developed the theory of mental accounting, explaining how people simplify financial decision-making by creating separate accounts in their minds, focusing on the narrow impact of each individual decision rather than its overall effect’.

**Table 1.** Problems reviewed in Thaler [1]: citations, descriptions and hypotheses*.*

**Pr**oblem	Google Scholar citations	description and hypothesis	explanation
1	Tversky and Kahneman,1986 [13] (790)	(**risk taking**) manipulation with two conditions testing diminishing sensitivity towards gain and loss. **H1:** people are risk-averse for gains and risk-seeking for losses.	People perceive outcomes according to the value function in prospect theory. Changes in wealth (gains and losses), rather than wealth levels, are effect carriers of values.
2	Tversky and Kahneman,1981 [6] (28623)	(**time investment versus price reference point**) manipulation with two conditions testing reference points. **H2:** people are more likely to spend 20 minutes to save $5 out of $15 than to save $5 out of $125.	People may frame outcomes in terms of a topical account, where the consequences of possible choices are related to a reference level that is determined by the decision’s context. And these reference points can shift evaluations of value.
3	Tversky and Kahneman, 1981 [6] (28623)	(**theatre play ticket**) manipulation with two conditions testing the impact of an existing account on decisions. **H3:** not explicitly reported. **reconstructed hypothesis:** people are more willing to buy a ticket when they have lost an equivalent amount of cash than when they lost their ticket (different mental accounts).	People may evaluate decisions in a more inclusive account when the outcomes of the act can influence the balance in an account that was previously established by a related act. In general, the sunk cost effect occurs when the decision is referred to an existing account with a negative current balance.
4	Thaler, 1985 [9] (9860)	(**events and happiness**) four pairs of scenarios testing the hedonic framing. **H4:** people follow four principles (a) segregate gains, (b) integrate loss, (c) cancel smaller losses against larger gains and (d) segregate ‘silver linings’ (small gains) from larger losses.	People tend to frame outcomes or code combinations of events in ways that make them the happiest.
5	Thaler and Johnson, 1990 [8] (3673)	(**same day or two weeks apart**) three pairs of events testing the temporal spacing of hedonic editing. **H5:** people choose to have the events occur ‘apart’ when segregation is preferred, and ‘together’ when integration is hedonically optimal.	People tend to simplify and encode multiple outcomes in a hedonically optimal manner. The temporal separation will facilitate cognitive segregation, whereas the temporal proximity will facilitate cognitive integration.
6	Thaler and Johnson, 1990 [8] (3673)	(**emotional impact of losing $9**) manipulation with two conditions testing the effect of a prior loss. **H6:** not explicitly reported. **reconstructed hypothesis:** people integrate subsequent losses with prior gains but not with prior losses. Also, people are not very sensitive to the exact value of the prior losses when they are within the same magnitude as subsequent losses.	People only follow the hedonic editing rules for part of the time (the quasi-hedonic editing hypothesis).
7	Thaler, 1985 [9] (9860)	(**location and price**) manipulation with two conditions testing the impact of context reference points on willingness to pay. **H7:** people are willing to pay more for the same product if bought from a resort than if bought from a grocery store.	Consumption decisions are influenced by people’s reference points as set by the context (resort is perceived as a higher reference price than that of a grocery store).
8	Thaler, 1985 [9] (9860)	(**selling ticket**) manipulation with three conditions testing the determinants of the reference points. **H8:** not explicitly reported. **reconstructed hypothesis:** people request a price equal to cost when selling to a friend and a price equal to market price when selling to a stranger, unless their cost exceeds market price.	Fairness, which largely depends on the cost to the seller, is the dominant factor in determining reference price.
9	Shafir and Thaler, 1998, unpublished data^a^ (8)	(**wine bottle**) manipulation with two conditions testing the value of wine. **H9:** not explicitly reported. **reconstructed hypothesis:** people exhibit a lack of consensus regarding the perception of the cost.	People hold mixed perceptions of the value of items when the consumption and purchase are temporally separated.
10	Shafir and Thaler, 1998, unpublished data (8)	(**feelings about purchase**) three statements examining ‘investment’ purchases. **H10:** not explicitly reported. **reconstructed hypothesis:** people are more agreeable with the view that the wine purchase is an investment.	For purchases to be consumed in the distant future, people may perceive the expense as an ‘investment’, thereby avoiding the feeling of spending.
11	Heath and Soll, 1996 [3]^b^ [Study 2]	(**previous events and new payment**) manipulation with two conditions testing the underconsumption of a typical target in three contexts, two unrelated and one related. **H11a:** the budget-setting process promotes greater underconsumption in the $50 condition than the $20 condition. **H11b:** the expense-tracking process promotes greater underconsumption for related purchases.	People set budgets for different accounts and recomputed the remaining budgets periodically. They will decrease further expenses for related activities but less so for unrelated activities.
12	Leclerc et al., 1995 [15] (865)	(**the performance**) manipulation with two conditions testing whether the value of time is influenced by price-related characteristics of a decision situation. **H12:** subjects are willing to pay more money to avoid waiting the same amount of time for a higher-priced food or service than for a lower-priced product.	The value of time is influenced by contextual effects postulated by prospect theory.
13−15	Thaler, 1999 [1] (5912)	(**choices**) a gain (P13) versus loss (P14−P15) scenario examining prior outcomes and risky choices. **H13:** the ‘house money’ effect—prior gain stimulates risk seeking. **H14−H15**: weaker to no effects for prior loss unless the gamble offers a chance to break even.	When gambles are bracketed together, the outcome of the prior gamble can influence subsequent choices.
16	Samuelson, 1963 [16] (1102)	(**coin flip bet**) a scenario testing how bracketing gambles affects the attractiveness of individual bets. **H16**: people shift between single gambles and long-term repeating gambles.	People’s preference follows a piecewise linear version of the value function in prospect theory. One bet yields negative expected utility, while repetitive bets yield positive expected utility.
17	Thaler, 1999 [1] (5912)	(**division investment**) two scenarios examining the myopic loss aversion effect. **H17:** not explicitly reported. **reconstructed hypothesis:** people are less willing to undertake a single risky investment, but more willing to undertake a portfolio of 25 investments.	Narrow framing inhibits risk-taking, but this can be avoided by aggregation across time or across different divisions.

Note. The Google Scholar citations were noted in May 2025.

^a^ For Shafir and Thaler, 1998, unpublished data [14], it was later published titled ‘Invest now, drink later, spend never: the mental accounting of delayed consumption’ and there were 266 Google Scholar citations.

^b^ Thaler [1] referred to Heath and Soll’s [3] example of mental accounting of ‘sports game’ and ‘parking ticket’ as different accounts. Sample items and statistics were not provided for that example in Heath and Soll [3], and so we tested it using the example that was provided regarding sports and theatre tickets as related accounts, and dinner, flu inoculation and theatre tickets as separate accounts.

We also recognized the potential for updating and improving both the transparency and the methods used in some of the problems reviewed by Thaler [1]. For example, among the problems we aimed to replicate, several did not report basic methodological details like sample size or descriptives. Statistical analyses were also often not conducted or not reported in sufficient detail for reproducibility. These challenges suggest the need to revisit these problems to reproduce their materials, deduce and improve on their methods, and reassess and update their findings to meet current-day best practices.

In sum, we aimed to revisit the classic mental accounting phenomenon to examine the reproducibility and replicability of the findings with replications by an external independent team. Following the recent growing recognition of the importance of reproducibility and replicability in psychological science (e.g. [17,18]), we embarked on a well-powered pre-registered very close replication of the work reviewed by Thaler [1].

We note that when we embarked on this Registered Report, there were no published systematic attempts for direct replications of the mental accounting findings reviewed in Thaler [1], and there were no published independent direct pre-registered well-powered replications of Thaler’s own work. During our work on revising Stage 2, a multi-country group [19] released the findings from their multi-country collaboration in which they revisited many mental accounting experiments, with results very similar to the ones we report below.

### 1.3. Thaler, 1999: original hypotheses

Thaler’s [1] review synthesized over a decade of research on the mental accounting phenomenon, and we aimed to focus on 17 classic problems he covered. We provided a summary of the original studies and their hypotheses in table 1.

For each of the replication problems, we followed the original experimental design with minor adjustments to make those suitable for our target sample (see §2.6). We then added four additional experiments to examine predictions Thaler [1] made that were not reviewed with supporting empirical evidence. We provided a full description of all the problems in the electronic supplementary material, section ‘Materials and scales used’.

### 1.4. Extensions: prediction extensions

We extended the replication by also adding a test of four predictions that the Thaler [1] review reflected on but did not provide empirical evidence that directly tested these predictions. We summarized our extensions in table 2.

**Table 2.** Extension: summary of predictions made by Thaler [1] with no reviewed supporting evidence.

extensions	description and predictions/hypothesis	explanation
Problem 18	Thaler, 1980 [12] **game in bad weather** two scenarios testing the sunk cost effect. **H18:** if the family pays for the tickets, they will tend to go despite the weather. If the tickets are given to them, they will tend to stay home.	Payment for a good increases the likelihood of its usage.
Problem 19	Thaler, 1980 [12] **membership and tennis elbow** one scenario examining the sunk cost effect. **H19:** purchasing membership will lead to continued play despite pain.	Paying for the right to use a service increases the likelihood of utilization.
Problem 20^a^	Thaler, 1999 [1] **price and decision** two statements testing how sunk costs affect subsequent decisions. **H20:** the more one paid for the shoes, the more times one will try to wear them. Eventually, one stops trying. But the more one paid, the longer one will keep the shoes before throwing them away.	The effect of sunk costs on subsequent decisions is not persistent.
Problem 21^b^	Thaler, 1999 [1] **annual membership** manipulations with three conditions testing expenses framing. **H21:** membership phrased as ‘merely 27 cents a day’ will be more attractive.	People tend to ignore small, routine expenses.

Note. The papers listed are the sources of the predictions yet none of the predictions have been tested directly to the best of our knowledge.

^a^ For Problem 20, we aimed to examine how much participants identify with Thaler’s [1] prediction.

^b^ The pennies-a-day effect in Problem 21 has been investigated in the marketing field (e.g. [20]).

### 1.5. Pre-registration and open science

We provided all materials, data and code on the Open Science Framework (OSF): https://osf.io/v7fbj/.

This Registered Report was submitted to the *Royal Society Open Science* following peer review and recommendation for Stage 2 acceptance at the *Peer Community In* (PCI) *Registered Reports*’ platform. Full details of the peer review and recommendation of the paper at PCI Registered Reports may be found at the links below. After submission to the journal, the paper received no additional external peer review but was accepted on the basis of the Editor’s recommendation according to the RSOS PCI Registered Reports’ policy (https://royalsocietypublishing.org/rsos/registered-reports#PCIRR). Stage 1 recommendation and review history: https://rr.peercommunityin.org/articles/rec?id=164 / https://osf.io/d6cjk/ (our frozen pre-registration version of the entire Stage 1 packet: https://osf.io/xu7jb/). Stage 2 recommendation and review history: Chambers [21]; https://doi.org/10.24072/pci.rr.100375. All measures, manipulations and exclusions conducted for this investigation are reported, and data collection was completed before analyses. The project was part of a large mass replications and extensions project (CORE, 2025), which received ethics approval from the University of Hong Kong (no. EA210265). This Registered Report was written based on the Registered Report template by Feldman [22].

## 2. Method

### 2.1. Power analysis

To ensure that the replication would have sufficient power, we first calculated the effect sizes of the original studies based on the statistics reported. Then, power analysis was conducted with a setting of alpha (two-sided) = 0.05 and power = 0.95. The calculation of effect size and power was with the help of a guide by Jané *et al.* [23] and R (version 4.3.1 [24]; using packages ‘MBESS’ and ‘pwr’ [25,26]). The largest required sample size was 321 participants, indicated by the power analysis of Problem 15. We provided more information regarding these calculations in the subsection ‘Power analysis of original study effect to assess required sample for replication’ in the electronic supplementary material and Rmarkdown code provided in the OSF folder.

Given the possibility that the original effects are overestimated, and taking into account the issues of multiple comparisons and potential exclusions, we aimed to recruit 500 participants. Given reviewer feedback in Stage 1 regarding the possibility of participant fatigue and the long survey duration, we made a change to our implementation so that each participant was randomized into 9 of the 18 Qualtrics blocks, cutting survey time on average by half. To compensate for that, we doubled our overall target sample to 1000. A sensitivity analysis indicated that a sample of 500 allows the detection of effects of *f* = 0.17 (groups = 3, d.f. = 1) and *d* = 0.29/0.36 (between, 250/166 in each condition) (both 95% power, alpha = 5%, one-tail), which are effects much weaker than any of the supported effects in the reviewed studies.

### 2.2. Participants

We recruited native English speakers who were born, raised and located in the United States on Amazon Mechanical Turk using the CloudResearch platform [27]. A total of 1007 participants completed the study (*M*_age_ = 43.28, s.d. = 12.61; 471 females, 526 males, 3 others and 7 rather not disclose). As participants were randomized to complete 9 out of 18 Qualtrics blocks, there were approximately 500 participants for each problem. We note that 1073 subjects began the survey, but 66 did not proceed beyond the consent and verifications. We summarized the sample differences between the current replication and the original studies in table 3.

**Table 3.** Original studies reviewed in Thaler [1]: summary of samples.

factors	sample size	characteristics	medium and compensation
Current replication	Total: 1007; Problem: ~500	US American (median age = 40.00 years, average age = 43.28 years, standard deviation age = 12.61 years, age range = 20–80 years)	online through compute
Problem 1	254	126 for Gain condition, 128 for Loss condition	unreported
Problem 2	181	93 for $15 calculator condition, 88 for $125 calculator condition	unreported
Problem 3	383	183 for ‘Lost a bill’ condition, 200 for ‘Lost the ticket’ condition	unreported
Problem 4	87	undergraduate students in a statistical class at Cornell University	in person
Problem 5	65	—	unreported
Problem 6	137	Cornell UG students: 137 for condition A 1−3	unreported
	168	Cornell MBA students, 87 for condition A and 81 for condition B	
Problem 7	unreported	regular beer drinkers in an executive development program	in person
Problem 8	85	first-year MBA students, 31 for Free condition, 28 for Paid $5 condition, 26 for Paid $10 condition	unreported
Problem 9	173	subscribers to a wine newsletter, *Liquid Assets*, and are highly knowledgeable wine consumers with substantial home cellars, 97 for Giving away condition and 76 for Drinking condition	unreported
Problem 10	unreported	subscribers to a wine newsletter, *Liquid Assets*	unreported
Problem 11	66	MBA students, split evenly across conditions	in person, pizza and beer
Problem 12	67	MBA students, 37 male and 30 female	unreported
Problem 13	unreported	MBA students	in person
Problem 14	unreported	MBA students	in person
Problem 15	unreported	MBA students	in person
Problem 16	1	an economist colleague	in person
Problem 17	26	a CEO and 25 executives from one firm, each managing a separate division	in person

We used the following CloudResearch options: Duplicate IP Block, Duplicate Geocode Block, Suspicious Geocode Block, Verify Worker Country Location, Enhanced Privacy, CloudResearch Approved Participants, Block Low Quality Participants, etc. We first pretested survey duration with 30 participants to test time run estimate and adjusted pay based on the duration. As pre-registered, the data of the 30 participants was included in the final data analysis but was not analysed independently other than to assess survey completion duration and needed pay adjustments.

### 2.3. Design

We summarized the experimental designs in table 4. We mapped the designs used in the problems, which included one-sample, between-subject, within-subject and mixed experimental designs. We set up all the problems using Qualtrics. We had a total of 18 Qualtrics blocks, and empirically related problems were grouped in a single Qualtrics block so that the same participants answered all related problems: (i) Problems 13, 14 and 15, and (ii) Problems 18 and 19.

**Table 4.** Replications and extensions of experimental designs.

problem design	independent variables	dependent variables
Problem 1: between	**gain condition:** choices between sure/uncertain *gain* **loss condition:** choices between sure/uncertain *loss*	*risk-taking preference (choice):* risk taking versus risk averse
Problem 2: between	**$15 calculator condition:** jacket is $125, calculator is $15 **$125 calculator condition:** jacket is $15, calculator is $125	*willingness to travel to another store (choice*): yes versus no
Problem 3: between	**‘Lost a bill’ condition:** lost a $10 bill as you enter the theatre **‘Lost the ticket’ condition:** lost the $10 ticket as you enter the theatre	*willingness to buy (another) ticket (choice):* yes versus no
Problem 4: one sample proportions	hedonic framing	*emotionally equivalence* (*choice*): participants indicate who was happier/more upset
Problem 5: within	temporal spacing	*emotionally equivalence (choice*): participants indicate who was happier/more unhappy
Problem 6: between	incremental impact of loss manipulation: different prior outcomes	*emotional impact of losing $9 (choice*): participants indicate which event hurts more
Problem 7: between	**hotel condition:** the soda is sold at a fancy resort hotel **grocery store condition**: the soda is sold at a small, run-down grocery store	*price willing to pay (continuous)*
Problem 8: mixed	**cost** (between): free versus $5 versus $10 **buyer** (within): friend versus strange **market price** (within): $5 versus $10	*price willing to sell (continuous*): participants indicate their selling price when the customer is a friend/stranger when the going price is $5/$10
Problem 9: between	**drinking condition:** participants are to imagine drinking a bottle of the wine with dinner **giving away condition:** participants are to imagine giving one bottle of the wine to a friend as a gift	*feeling of the cost (choice):* participants indicate which statement best captures their feelings regarding the cost
Problem 10: within	purchase of Bordeaux futures at $400	*feeling about the purchase (ordinal*) participants indicate which statement best captures their feelings about the purchase
Problem 11^a^: mixed	**between**: $50 versus $20 **within** (5 conditions): dinner-spent versus dinner-given versus ticket-spent versus ticket-given versus flu	*willingness to buy a $25 theatre ticket later in the week (choice)*
Problem 12^b^: between	**$15 condition:** the tickets will cost $15 each **$40 condition:** the tickets will cost $40 each	*price willing to pay to avoid waiting (continuous)*
Problem 13^c^: single (within: compared against 14, 15)	‘House money’ won $30 scenario—loss potential: risk-seeking versus risk-averse	*risk taking (choice):* participants imagine winning $30, then choose between uncertain gain/loss or no further gain/loss
Problem 14: single (within: compared against 13, 15)	lost $30—loss potential: risk-seeking versus risk-averse	*risk taking (choice*): participants imagine losing $30, then choose between uncertain gain/loss or no further gain/loss
Problem 15: single (within: compared against 13, 14)	lost $30—no loss potential (gain): risk-seeking versus risk-averse	*risk taking (choice*): imagine losing $30, participants then choose between uncertain gain or a sure gain
Problem 16: within	coin flip bet	*willingness to take the bet (choice*): decision under a single coin flip/100 coin flips
Problem 17: within	division investment	*willingness to undertake the investment (choice*): decision under a single project/a portfolio of 25 projects
Problem 18^d^ (extension): within	the cost of the ticket	*willingness to go to the game (choice*): decision between go to the game and stay home when the ticket is bought/given
Problem 19 (extension): within	membership at tennis club	*willingness to play (choice*): decision between stop playing and continue playing
Problem 20 (extension): within	shoe purchase scenario	*sunk cost effects* ***(*** *continuous*): participants are to indicate how accurately the statements apply to them
Problem 21 (extension): between	**day expression condition:** merely 27 cents a day **year expression condition:** 100 US$ a year **Both expressions condition:** merely 27 cents a day versus 100 US$ a year	*attractiveness of the membership plan (continuous)*

Note.We detailed the options of each problem in table 5 and table 6.

^a^ For Problem 11, Thaler [1] did not summarize the study design comprehensively, and we also found the method of the original article difficult to understand. Therefore, we only replicated part of Study 2 in Heath and Soll [3].

^b^ For Problem 12, it is possible that Thaler [1] wrongly reported the second condition, as our understanding is that the ticket price should be $40, whereas Thaler [1] wrote $45. For our replication, we followed our understanding of the original version.

^c^ Problems 13, 14, and 15 were in a single Qualtrics block (grouped together, random order; within-subject design).

^d^ Problems 18 and 19 were in a single Qualtrics block (grouped together, random order; within-subject design).

In order to address reviewer’s feedback in Stage 1 to decrease the length of the survey and the cognitive burden on participants, we randomly assigned participants to complete 9 of the 18 blocks. The display of problems and conditions was counterbalanced using the randomizer ‘evenly present’ function in Qualtrics.

We previously tested including many problems in a single data collection using a unified design in many other replications and extensions conducted by our team, and our experience has shown that combining several experiments in a single unified data collection in random order does not seem to impact the likelihood of replication success. For example, we successfully ran a similar design in our replications of studies reviewed in influential review papers by Kahneman and Tversky [28] (Mayiwar *et al*., [29]), Read *et al*. [30] (Wong & Feldman, [31]), Heath *et al.* [32] (Au & Feldman, [33]) and Tversky and Kahneman [34] (Hong & Feldman [35]). This design seems especially powerful in addressing concerns about the target sample (naivety, attentiveness, etc.) when some studies replicate successfully whereas others do not.

### 2.4. Procedure

Participants first read a consent form and indicated their willingness to participate, and then answered several verification questions. Three questions assessed their eligibility, understanding and agreement with study terms, which they had to answer with a ‘yes’ and the required responses to proceed to the study. The three questions also served as attention checks, with a randomized display order of the options—(i) ‘Are you able to pay close attention to the details provided and carefully answer questions that follow?’ (yes/no/not sure), (ii) ‘Do you understand the study outline and are willing to participate in a survey with comprehension checks?’ (yes/no/not sure), and (iii) ‘Are you a native English speaker born, raised and currently located in the United States?’ (yes/no). Failing any of the three questions meant that the participants did not indicate consent and therefore could not embark on the study. Upon completion of these steps, participants proceeded to begin the survey.

Participants were then randomly assigned to answer problems in 9 of the 18 Qualtrics blocks. At the end of the survey, participants answered funneling questions and provided their demographic information before being directed to the debriefing.

### 2.5. Manipulations and measures

We summarized all problems and manipulations in table 4. We summarized the measures and data analysis strategies for all replication problems in table 5. We added four problems that were not originally tested in the review article, and we summarized the measures and data analysis strategies for these extensions in table 6.

**Table 5.** Replication problems: measures and data analysis strategies.

Problem	measure	data analysis strategy	
		in the original	deduced additional analysis
1	choose between two choices (displayed in random order)	calculated the cumulative per cent frequency for each choice	Chi-square
2	answer the Yes/No question (options displayed in random order)	calculated the cumulative per cent frequency for the Yes option	Chi-square
3	answer the Yes/No question (options displayed in random order)	calculated the cumulative per cent frequency for each choice	Chi-square
4	four pairs of scenarios are presented in random order. Choose among three choices	calculated the cumulative per cent frequency for all three choices	proportion tests
5	three pairs of scenarios are presented in random order. Choose among three choices.	calculated the cumulative per cent frequency for all three choices	proportion tests McNemar paired-sample tests: A–B A–C
6	five pairs of events displayed in random order. Choose among three choices	calculated the cumulative per cent frequency for all three choices	proportion tests
7	report what price they will tell the friend	calculated the median for the two conditions	independent-samples *t*‐test
8^a^	report what price they will ask under different conditions	calculated per cent of subjects giving common answers (0, 5, 10, other)	mixed ANOVA: 3 between: free versus paid $5 versus paid $10 2 within: friend versus stranger 2 within: market worth $5 versus $10
9	choose among five statements (displayed in random order)	calculated the cumulative per cent frequency for all five statements	Chi-square
10	indicate which statement more accurately captures their feelings on a 5-point Likert scale (four statements displayed in random order)	calculated the mean score of the statements	repeated measures ANOVA
11	five scenarios are presented in random order. Answer the Yes/No question	calculated the correlation between underconsumption and typicality	mixed ANOVA: 2 between: $20 low-cost versus $50 high-cost 2 within: given versus spent 2 within: dinner versus ticket
12	report how much they would be willing to pay to avoid waiting	calculated the mean score for each condition and conducted independent-sample *t*-tests	subtract the price of the ticket, exclude data below 0 and conduct independent-samples *t*‐test
13	choose between two choices (displayed in random order)	calculated the cumulative per cent frequency for each choice	baseline against 14 and 15
14	choose between two choices (displayed in random order)	calculated the cumulative per cent frequency for each choice	McNemar paired-sample tests 13−14
15	choose between two choices (displayed in random order)	calculated the cumulative per cent frequency for each choice	McNemar paired-sample tests 13−15
16	two scenarios are presented in random order. Answer the Yes/No question	no data analysis was performed	McNemar paired-sample tests
17	two scenarios are presented in random order. Answer the Yes/No question	no data analysis was performed	McNemar paired-sample tests

^a^ For Problem 8, there was no explanation provided regarding the classification of ‘common answers’, so we can only assume that any value other than 0, 5 and 10 were counted as ‘Other’.

**Table 6.** Measures and data analysis strategies for prediction extension problems.

Problem	measure	data analysis strategy
18	choose between two choices (displayed in random order)	McNemar paired-sample tests
19	choose between two choices (displayed in random order)	proportions test
20	report how accurately the two statements express their feelings on a 5-point Likert scale	one-sample *t*‐test against the midpoint
21	rate the attractiveness of the membership plan on a 0−100 scale	independent-sample *t*‐test paired sample *t*‐test

We provided full details of the manipulations between the conditions and the experimental designs in the supplementary materials section ‘Materials and scales used in the replication+extension problems’. Problems 1–3, 6–9, 11, 12 and 21 involved between-subjects manipulations, and participants were randomly assigned to conditions separately in each of those. The order of the problems was also randomized.

### 2.6. Deviations from the original studies

Our replication deviations from the target article’s studies include participants’ characteristics, delivery mode and the extensions. We summarized our adjustments and deviations in tables 7 and 8.

**Table 7.** Deviations in replications compared with target studies.

Problem	deviation details	reasons for change
1	We adopted the wording Thaler [1] used in his work. For the Gain condition, the original second option was framed as ‘*A 50% chance to gain $200 and a 50% chance to lose $0*’. We changed it to ‘*A 50% chance to gain $200 and a 50% chance to gain $0*’ in our current replication.	We aimed to be as close as possible to the target article. While comparing the option with the Loss condition, we suspected it as a typo.
4	We slightly simplified the description of the problem and randomized the sequence of the scenarios.	Minor wording changes for clarity. Randomization to eliminate order bias.
5, 6	We revised and simplified the description of the problems and the options. We also randomized the sequence of the scenarios.	Minor wording changes for clarity. Randomization to eliminate order bias.
7	The original study used beer in the two conditions, and we changed it to soda.	Some of our targeted participants may not drink.
9	Added ‘Imagine that you enjoy drinking wine’ at the beginning of the scenario and randomized the sequence of the statements.	Our targeted population might not enjoy drinking wine. Randomization to eliminate order bias.
10	(1) Added ‘Imagine that you enjoy drinking wine’ at the beginning of the scenario (2) Added another option ‘*I cannot understand this question*’ (3) Changed the Likert scale to a 1 (*not accurate at all*) to 5 (*very accurate*) rating (4) Randomized the sequence of the statements.	(1) Our targeted population might not enjoy drinking wine. (2) Our pretest showed that this scenario might be too vague and difficult to comprehend for our targeted participants, so we added another statement to check for understanding. By adding this option, we ensured that participants do not just choose a random option when they cannot understand the question. (3) To reduce cognitive load. (4) Randomization to eliminate order bias.
11	We simplified the instructions for the problem.	Minor wording changes for clarity and understandability.
12	The original study used student tickets at the student window in the scenarios. We changed it into discounted tickets and discount windows. The question is revised.	Our targeted population would have a wide age range from 18 to 80, so many of them might not be students. Our pretest showed that the original framing of the question caused misunderstandings. We revised for greater clarity.
13, 14, 15	Added ‘Imagine that…’ at the beginning of the scenarios.	To facilitate perspective-taking.
21 (extension)	Thaler [1] used ‘local public radio station’ in his prediction, while we changed it into ‘music online streaming service’.	The original scenario does not apply to 2022 so we change it to update to current time.

**Table 8.** Classification of the replication, based on LeBel *et al*. [5].

design facet	replication	details of deviation
effect/hypothesis	same	
IV construct	same	
DV construct	same	
IV operationalization	same	
DV operationalization	same	
IV stimuli	similar	Scenarios were slightly adjusted to update to current time and the targeted population.
DV stimuli	similar	Problem 1 was changed for a suspicious typo, and we added another statement in Problem 10.
procedural details	similar/ different	We combined all studies into a singular unified design; we randomized the order of scenarios/statements in the problems.
physical settings	different	The current replication was conducted online through Qualtrics.
population (e.g. age)	different	We collected data online from MTurk using CloudResearch.
contextual variables	different	
replication classification	very close replication	Based on the above analysis, we summarized our replications as a ‘very close’ replication of the original studies.

Note. IV refers to independent variable, and DV refers to dependent variable.

### 2.7. Evaluation criteria for replication findings

We aimed to compare the replication effects with the effects in the original studies using the criteria set by LeBel *et al*. [36].

### 2.8. Replication closeness evaluation

We provided details on the classification of the replications using the criteria by LeBel *et al*. [5] in table 8 (also see section *‘*Replication Evaluation’ in the electronic supplementary material). We summarized the replication as a ‘very close’ replication.

### 2.9. Exclusions

We did not exclude participants, following our pre-registered plan to focus on the main sample, and so in our analysis, we included all the data of those who successfully completed the entire study.

## 3. Results participants did not choose the correct answer

We conducted our analyses on the full sample (pre-registered) using R (version 4.3.2) [24] and the JAMOVI ‘jmv’ [37].

In tables 9–11, we summarized the descriptives for all the problems, alongside the findings from the original studies to allow for an easy comparison. We summarized the statistical tests in tables 12–15. We compared the original studies’ effects to ours in table 16.

**Table 9.** Descriptive statistics for choice problems.

Problem	conditions and options	replication			original	replication interpretation
		*n*	count	percentage	percentage	
1	**gain:**	250				successful
	a sure gain of $100		209	84%	72%	
	a 50% chance to gain $200 and a 50% chance to gain $0		41	16%	28%	
	**loss:**	254				
	a sure loss of $100		112	44%	36%	
	a 50% chance to lose $200 and a 50% chance to lose $0		142	56%	64%	
2	**$15 calculator:**	253				*unsuccessful, no signal* original showed a reversal. Replication no difference and generally low rates of willingness to make the trip.
	make the trip		29	11%	68%	
	not making the trip		224	89%	32%	
	**$125 calculator:**	252				
	make the trip		17	7%	29%	
	not making the trip		235	93%	71%	
3	**lost a $10 bill:**	252				successful
	buy the ticket		228	90%	88%	
	not buying the ticket		24	10%	12%	
	**lost the ticket:**	251				
	buy another ticket		194	77%	46%	
	not buying another ticket		57	23%	54%	
4	**A. two wins: $50, and $25 versus one win: $75**	505				successful
	two wins is happier		178	35%	64%	
	one win is happier		62	12%	18%	
	no difference		265	52%	17%	
	**B. two mistakes: $100, and $50 versus one mistake: $150**	505				successful
	two mistakes is more upset		213	42%	76%	
	one mistake is more upset		69	14%	16%	
	no difference		223	44%	8%	
	**C. two events: win $100, and loss $80 versus One event: win $20**	505				successful
	two events is happier		61	12%	25%	
	one event is happier		386	76%	70%	
	no difference		58	11%	5%	
	**D. two events: loss $200, and win $25 versus one event: loss $175**	505				successful
	two events is more upset		63	12%	22%	
	one event is more upset		316	63%	72%	
	no difference		126	25%	6%	
5	**A. two events: (1) win $25 (2) win $50**	495				*unsuccessful, opposite*
	happier on the same day		200	40%	25%	
	happier two weeks apart		174	35%	63%	
	no difference		121	24%	12%	
	**B. two events: (1) $100 must be paid (2) $50 must be paid**	495				successful
	more unhappy on the same day		303	61%	57%	
	more unhappy two weeks apart		96	19%	34%	
	no difference		96	19%	9%	
	**C. two events: (1) a $20 parking ticket (2) a $25 bill**	495				successful
	more unhappy on the same day		278	56%	75%	
	more unhappy two weeks apart		115	23%	17%	
	no difference		102	21%	7%	
6^a^	**First group of questions:**					
	**1. (A) lose $9 (B) lose $9 after having gained $30**	253				successful
	A hurts more		208	82%	70% / 84%	
	B hurts more		32	13%	9% / 10%	
	no difference		13	5%	21% / 6%	
	**2. (A) lose $9 (B) lose $9 after having lost $30**					successful
	A hurts more		19	8%	13% / 22%	
	B hurts more		218	86%	55% / 75%	
	no difference		16	6%	31% / 3%	
	**3. (A) lose $9 (B) lose $9 after having lost $250**					*unsuccessful, opposite*
	A hurts more		32	13%	39% / 54%	
	B hurts more		209	*83%*	*38% / 37%*	
	no difference		12	5%	23% / 9%	
	**4. (A) lose $9 (B) lose $9 after suffering a loss of $1000**					*unsuccessful, opposite*
	A hurts more		37	15%	50%	
	B hurts more		201	79%	33%	
	no difference		15	*6%*	17%	
	**5. (A) lose $9 after suffering a loss of $30 (B) lose $9 after suffering a loss of $1000**					*unsuccessful, opposite*
	A hurts more		51	20%	51%	
	B hurts more		184	73%	38%	
	no difference		18	7%	*21%*	
	**Second group of questions:**					
	**1. (A) lose $9 (B) lose $9 after suffering a loss of $9**	252				successful
	A hurts more		9	4%	7%	
	B hurts more		209	83%	64%	
	no difference		34	13%	28%	
	**2. (A) lose $9 (B) lose $9 after suffering a loss of $18**					successful
	A hurts more		9	4%	11%	
	B hurts more		231	92%	65%	
	no difference		12	5%	23%	
	**3. (A) lose $9 (B) lose $9 after suffering a loss of $36**					successful
	A hurts more		11	4%	12%	
	B hurts more		228	90%	62%	
	no difference		13	5%	26%	
	**4. (A) lose $9 (B) lose $9 after suffering a loss of $45**					successful
	A hurts more		10	4%	14%	
	B hurts more		230	91%	65%	
	no difference		12	5%	21%	
	**5. (A) lose $9 after suffering a loss of $9 (B) lose $9 after suffering a loss of $36**					successful
	A hurts more		16	6%	7%	
	B hurts more		224	89%	68%	
	no difference		12	5%	25%	
9^b^	**Giving away:**	254				successful $75 is considered the correct answer, and both original and replication show most participants did not choose the correct answer.
	$0		75	30%	30%	
	$20		52	20%	17%	
	$20 plus interest		14	6%	9%	
	$75		53	21%	30%	
	A $55 saving		60	24%	14%	
	**Drinking:**	251				
	$0		57	23%	30%	
	$20		54	22%	18%	
	$20 plus interest		16	6%	7%	
	$75		64	25%	20%	
	A $55 saving		60	24%	25%	
11^c^	**$50 high cost:**	254				partially successful
	**spent $50 on dinner. Would you buy a $25 theatre ticket later in the week?**					
	buy the ticket		101	40%	—	
	not buying the ticket		153	60%	—	
	**given a $50 dinner. Would you buy a $25 theatre ticket later in the week?**					
	buy the ticket		211	83%	—	
	not buying the ticket		43	17%	—	
	**spent $50 on a sports ticket. Would you purchase a $25 theatre ticket later in the week?**					
	buy the ticket		70	28%	—	
	not buying the ticket		184	72%	—	
	**given a $50 sports ticket. Would you purchase a $25 theatre ticket later in the week?**					
	buy the ticket		198	78%	—	
	not buying the ticket		56	22%	—	
	**spent $50 on an inoculation. Would you purchase a $25 theatre ticket later in the week?**					
	buy the ticket		75	30%	—	
	not buying the ticket		179	70%	—	
	**$20 low cost:**	252				
	**spent $20 on dinner. Would you buy a $25 theatre ticket later in the week?**					
	buy the ticket		138	55%	—	
	not buying the ticket		114	45%	—	
	**given a $20 dinner. Would you buy a $25 theatre ticket later in the week?**					
	buy the ticket		213	85%	—	
	not buying the ticket		39	15%	—	
	**spent $20 on a sports ticket. Would you purchase a $25 theatre ticket later in the week?**					
	buy the ticket		103	41%	—	
	not buying the ticket		149	59%	—	
	**given a $20 sports ticket. Would you purchase a $25 theatre ticket later in the week?**					
	buy the ticket		201	80%		
	not buying the ticket		51	20%		
	**spent $20 on an inoculation. Would you purchase a $25 theatre ticket later in the week?**					
	buy the ticket		109	43%	—	
	not buying the ticket		143	57%	—	
13	**imagine that you have just won $30**	504				*unsuccessful*
	a 50% chance to gain $9 and a 50% chance to lose $9		143	28%	70%	
	no further gain or loss		361	72%	30%	
14	**imagine that you have just lost $30**	504				successful
	a 50% chance to gain $9 and a 50% chance to lose $9		85	17%	40%	
	no further gain or loss		419	83%	60%	
15	**imagine that you have just lost $30**	504				*unsuccessful*
	a 33% chance to gain $30 and a 67% chance to gain nothing		119	24%	60%	
	a sure $10		385	76%	40%	
16^d^	**a single-coin flip, heads you win $200, tail you lose $100**	506				successful
	take the bet		129	25%	—	
	not taking the bet		377	75%	—	
	**a package bet of 100 coin flips, each coin flip you either win $200 or lose $100**					
	take the bet		247	49%	—	
	not taking the bet		259	51%	—	
17^e^	**a project: 50% chance to gain $2 million, 50% chance to lose $1 million**	504				successful
	undertake the project		152	30%	12%	
	not undertaking the project		352	70%	88%	
	**a portfolio of 25 investments: each has a 50% chance of gaining $2 million and 50% chance of losing $1 million**					
	undertake the investments		236	47%	—	
	not undertaking the investments		268	53%	—	
18	**paid $40 for tickets:**	502			N/A	extension: supported
	go to the game		160	32%		
	stay home		342	68%		
	**tickets given by friends:**					
	go to the game		68	14%		
	stay home		434	86%		
19	**imagine that you joined a tennis club and paid a $300 yearly membership fee**.				N/A	extension: *not supported*
	stop playing	502	380	76%		
	continue to play		122	24%		

Note. *n* represents sample size per condition.

^a^ In Problem 6, Condition A−1, 2, 3 had two samples, so results are both presented, separated by a slash. The statistical results reported in Condition A−5 added up to 110% rather than 100%, suggesting a possible reporting mistake in the original article. For the calculation of the effect size we will assume ‘no differences’ is equal to 11% and not 21%.

^b^ In Problem 9, the mean for the ‘I don’t understand’ option was only 1.16, indicating that participants had a good understanding of the materials on average.

^c^ In Problem 11, Heath and Soll [3] revealed that a larger proportion of people are more likely to underconsume in the $50 high-cost than in the $20 low-cost condition (*t*(26) = 2.17, *p* < 0.05 by paired *t*‐test). The proportion of subjects who under-consume the target is highly correlated with typicality for both $50 high-cost (*r*(25) = 0.80, *p* < 0.01) and $20 low-cost conditions (*r*(25) = 0.67, *p* < 0.01). Yet we are unsure about the paired *t*‐test reported in Problem 11 as the experiment seems to adopt a between-subject design.

^d^ In Problem 16, the economist answered no for the single coin flip, and yes for playing the bet 100 times.

^e^ In Problem 17, 3 of the 25 executives accepted the single investment, and the CEO accepted the portfolio of 25 of these investments.

**Table 10.** Descriptive statistics for problems with scale/text entry and a between-subjects design.

Problem	condition			replication			original findings	replication interpretation
				*n*	mean	s.d.		
7	hotel (fancy) purchase			254	7.09	19.85	median = $5	successful
	grocery (run-down) purchase				4.17	3.98	median = $3	
8	free ticket	market value $5	friend	166	2.37	3.49	68% answer 0, 26% answer 5, 3% answer 10, and 3% answer other	successful
			stranger		6.04	5.24	6% answer 0, 77% answer 5, 10% answer 10, and 6% answer other	
		market value $10	friend		4.26	5.43	65% answer 0, 26% answer 5, 6% answer 10, and 3% answer other	
			stranger		10.32	6.79	6% answer 0, 16% answer 5, 58% answer 10, and 19% answer other	
	paid 5	market value $5	friend	169	3.72	2.47	14% answer 0, 79% answer 5, 0% answer 10, and 7% answer other	
			stranger		6.68	3.49	0% answer 0, 79% answer 5, 7% answer 10, and 14% answer other	
		market value $10	friend		6.12	4.20	7% answer 0, 79% answer 5, 4% answer 10, and 9% answer other	
			stranger		11.51	5.59	0% answer 0, 14% answer 5, 57% answer 10, and 29% answer other	
	paid 10	market value $5	friend	162	5.01	2.68	0% answer 0, 69% answer 5, 23% answer 10, and 8% answer other	
			stranger		8.19	3.58	0% answer 0, 42% answer 5, 46% answer 10, and 12% answer other	
		market value $10	friend		7.55	3.82	0% answer 0, 15% answer 5, 69% answer 10, and 15% answer other	
			stranger		11.43	4.01	0% answer 0, 0% answer 5, 73% answer 10, and 27% answer other	
12	$15 condition			235	8.14	11.32	people are willing to pay twice as much to avoid waiting for the $40 ticket than for the $15 ticket ( $\underset{\_}{X}$ =$7.20 versus $\underset{\_}{X}$ =$3.60, *t* = 1.92(39), *p* = .06).	successful
	$40 condition			222	10.34	7.66		
21	day expression			167	44.53	32.32	N/A	extension: supported
	year expression			166	26.04	28.05		
	both expressions		day	170	45.16	31.71		
			year		35.72	29.05		

Note. *n* represents sample size; s.d. represents standard deviation.

**Table 11.** Comparison and descriptive statistics for problems with scale and a within-subjects design.

Problem	options	replication			original findings	replication interpretation
		*n*	mean	s.d.		
10^a^	I feel like I just spent $400, much as I would feel if I spent $400 on a weekend getaway.	502	2.98	1.45	mean = 3.31	successful
	I feel like I made a $400 investment which I will gradually consume after a period of years.		3.56	1.30	mean = 1.94	
	I feel like I just saved $100, the difference between what the futures cost and what the wine will sell for when delivered.		3.08	1.36	mean = 2.88	
	I cannot understand this question.		1.16	0.65	—	
20	the more you paid for the shoes, the more times you will try to wear them.	507	3.10	1.41	N/A	extension: not supported
	eventually you stop wearing the shoes, but you do not throw them away. The more you paid for the shoes, the longer they sit in the back of your closet before you throw them away.		3.45	1.33		

Note. *n* represents sample size; s.d. represents standard deviation.

^a^ For Problem 10, the original study used the Likert Scale with a 1 (*strongly agree) to* 5 (*strongly disagree*) rating and the replication used a 1 (*not accurate at all*) to 5 (*very accurate*) rating.

**Table 12.** Problems 1−6, 9 and 19: Summary of **χ²** tests.

Problem	conditions and subquestions	*χ²*	d.f.	*p*
1	one-sample, single condition	85.03	1	<0.001
2	one-sample, single condition	3.39	1	0.066
3	one-sample, single condition	16.18	1	<0.001
4	A. two wins: $50 and $25 versus one win: $75.	123.24	2	<0.001
	B. two mistakes: $100 and $50 versus one mistake: $150	88.22	2	<0.001
	C. two events: win $100 and loss $80 versus one event: win $20	422.21	2	<0.001
	D. two events: loss $200 and win $25 versus one event: loss $175	206.10	2	<0.001
5	A. two events: (1) win $25 (2) win $50	19.65	2	<0.001
	B. two events: (1) $100 must be paid (2) $50 must be paid	173.13	2	<0.001
	C. two events: (1) a $20 parking ticket (2) a $25 bill	116.59	2	<0.001
6	first group of questions:			
	1. (A) lose $9 (B) lose $9 after having gained $30	274.16	2	<0.001
	2. (A) lose $9 (B) lose $9 after having lost $30	317.84	2	<0.001
	3. (A) lose $9 (B) lose $9 after having lost $250	278.81	2	<0.001
	4. (A) lose $9 (B) lose $9 after suffering a loss of $1000	244.96	2	<0.001
	5. (A) lose $9 after suffering a loss of $30 (B) lose $9 after suffering a loss of $1000	183.14	2	<0.001
	second group of questions:			
	1. (A) lose $9 (B) lose $9 after suffering a loss of $9	282.74	2	<0.001
	2. (A) lose $9 (B) lose $9 after suffering a loss of $18	385.93	2	<0.001
	3. (A) lose $9 (B) lose $9 after suffering a loss of $36	370.31	2	<0.001
	4. (A) lose $9 (B) lose $9 after suffering a loss of $45	380.67	2	<0.001
	5. (A) lose $9 after suffering a loss of $9 (B) lose $9 after suffering a loss of $36	350.10	2	<0.001
9	giving away	333	4	<0.001
	drinking	298	4	<0.001
	giving away versus drinking	3.64	4	0.457
19	imagine that you joined a tennis club and paid a $300 yearly membership fee	132.60	1	<0.001

Note. d.f. indicates degree of freedom

**Table 13.** Problems 5, 13−18: summary of paired-samples McNemar tests.

Problem	comparisons	*χ²*	d.f.	*p*	Cohen’s g
5^a^	comparing A to B A: (1) win $25 (2) win $50. Who is happier? B: (1) $100 must be paid (2) $50 must be paid. Who is more unhappy?	46.74	3	<0.001	—
	comparing A to C A: (1) win $25 (2) win $50. Who is happier? C: (1) a $20 parking ticket (2) a $25 bill. Who is more unhappy?	38.78	3	<0.001	—
13−14	Problems 13 versus 14 won $30 loss potential versus lost $30 loss potential	22.73	1	<0.001	0.20 [0.12, 0.26]
14−15	Problems 14 versus 15 (exploratory) lost $30 loss potential versus lost $30 *no loss* potential	8.38	1	0.004	0.12 [0.04, 0.20]
13−15	Problems 13 versus 15 won $30 loss potential versus lost $30 *no loss* potential	3.27	1	0.070	0.07 [-0.01, 0.14]
16	1 bet versus 100 bets	84.90	1	<0.001	0.36 [0.30, 0.40]
17	1 investment versus 25 investments	45.82	1	<0.001	0.27 [0.20, 0.33]
18	paid $40 versus given by friends	86.37	1	<0.001	0.47 [0.41, 0.49]

Note. d.f. indicates degree of freedom.

^a^ Problem 5 compared the same day to two weeks apart, higher same day for negative than for positive.

**Table 14.** Problems 7, 12, 20 and 21: summary of all *t*-tests results.

Problem	test type	statistic	d.f.	*p*	mean difference	s.e. difference	effect size	95% CI
7	independent-sample *t*‐test	Student’s *t* = 2.30	506	0.011	2.92	1.27	0.20	[0.03, 0.38]
		Welch’s *t* = 2.30	273.35	0.011	2.92	1.27	0.20	—
12	independent-sample *t*‐test	Student’s *t* = 2.42	455	0.016	2.20	0.91	0.23	[0.04, 0.41]
		Welch’s *t* = 2.45	412.97	0.015	2.20	0.90	0.23	—
20	one-sample *t*‐test	Statement 1: Student’s *t* = 1.64	506.00	0.051	0.10	—	0.07	[-0.01, 0.16]
		Statement 2: Student’s *t* = 7.53	506.00	<0.001	0.45	—	0.33	[0.24, 0.42]
21	independent-sample *t*‐test	Student’s *t* = 5.57	331	<0.001	18.48	3.32	0.61	[0.39, 0.83]
		Welch’s *t* = 5.57	325.07	<0.001	18.48	3.32	0.61	—
	paired sample *t*‐test	Student’s *t* = 3.82	169	<0.001	9.44	2.47	0.29	[0.14, 0.45]

Note. d.f. indicates degree of freedom, s.e. indicates standard error, and CI indicates confidence interval. Effect size for independent-sample *t-*test is Cohen’s *d*, effect size for paired sample *t*‐test is Cohen’s *dz*.

**Table 15.** Problems 8, 10, 11: Summary of all ANOVA results.

Problem	test type	source of variation	SS	d.f.	MS	*F*	*p*	*η²*
8	mixed ANOVA	friend versus stranger	8718.31	1	8718.31	594.75	<0.001	0.16
		market worth $5 versus $10	5072.36	1	5072.36	870.07	<0.001	0.09
		free ticket versus $5 versus $10	1736.17	2	868.08	15.69	<0.001	0.03
		friend versus stranger × free ticket versus $5 versus $10	146.59	2	73.29	5.00	0.007	0.00
		friend versus stranger × market worth $5 versus $10	418.89	1	418.89	205.04	<0.001	0.01
		market worth $5 versus $10 × free ticket versus $5 vs. $10	47.91	2	23.95	4.11	0.017	0.00
		friend versus stranger x market worth $5 versus $10 x free ticket versus $5 versus $10	79.70	2	39.85	19.50	<0.001	0.00
10	repeated measures ANOVA	accuracy of feelings	98.08	2	49.04	25.26	<0.001	0.03
11	mixed ANOVA	given versus spent	83.36	1	83.36	382.59	<0.001	0.17
		dinner versus ticket	4.09	1	4.09	55.52	<0.001	0.01
		high cost versus low cost	3.15	1	3.15	7.37	0.007	0.01
		given versus spent × high cost versus low cost	1.98	1	1.98	9.11	0.003	0.00
		given versus spent × dinner versus ticket	0.83	1	0.83	16.49	<0.001	0.00
		dinner versus ticket × high cost versus low cost	0.01	1	0.01	0.08	0.783	0.00
		given versus spent × dinner versus ticket x high cost versus low cost	0.01	1	0.01	0.26	0.610	0.00

Note. SS represents Type 3 sums of squares, d.f. represents degree of freedom and MS represents mean square

**Table 16.** Comparison of replication and original findings.

Problem	condition/subquestions	effect size	original e.s. [95% CI]	replication e.s. [95% CI]	interpretation
1	gain condition	*h*	0.46 [0.28, 0.63]	0.75 [0.62, 0.87]	
	loss condition	*h*	0.28 [0.11, 0.46]	0.12 [-0.00, 0.24]	
	**overall: two conditions compared**	**Cramer’s V**	**0.44 [0.32, 0.57]**	**0.41 [0.32, 0.50]**	**successful: signal-consistent**
2	$15 calculator condition	*h*	0.37 [0.17, 0.57]	-0.89 [-1.02, -0.77]	
	$125 calculator condition	*h*	0.43 [0.22, 0.64]	1.04 [0.91, 1.16]	
	**overall: two conditions compared**	**Cramer’s V**	**0.38 [0.24, 0.54]**	**0.07 [0.00, 0.17]**	**unsuccessful: no signal, inconsistent**
3	lost a $10 bill condition	*h*	0.86 [0.72, 1.01]	0.93 [0.80, 1.05]	
	lost the ticket condition	*h*	0.08 [-0.06, 0.22]	-0.57 [-0.69; -0.45]	
	**overall: two conditions compared**	**Cramer’s V**	**0.44 [0.34, 0.54]**	**0.17 [0.09, 0.27]**	**successful: signal-inconsistent, smaller**
4	4A segregate gains	Cramer’s V	0.29 [0.15, 0.45]	0.26 [0.20, 0.33]	signal-consistent
	4B integrate loss	Cramer’s V	0.42 [0.28, 0.58]	0.23 [0.17, 0.29]	signal-inconsistent, smaller
	4C cancel losses against larger gains	Cramer’s V	0.41 [0.27, 0.57]	0.43 [0.37, 0.49]	signal-consistent
	4D segregate small gains from larger losses	Cramer’s V	0.41 [0.27, 0.57]	0.31 [0.25, 0.37]	signal-inconsistent, smaller
	**overall**				**successful: 4 supported**
5	5A prefer segregation—happier two weeks apart	Cramer’s V	0.30 [0.13, 0.48]	0.09 [0.03, 0.16]	signal-inconsistent, opposite
	5B prefer integration—more unhappy two weeks apart	Cramer’s V	0.29 [0.13, 0.48]	0.28 [0.21, 0.34]	signal-consistent
	5C prefer integration—more unhappy two weeks apart	Cramer’s V	0.42 [0.26, 0.60]	0.23 [0.17, 0.29]	signal-inconsistent, smaller
	**overall**				**mixed: 2 supported, 1 unsupported-opposite**
6	*First group of questions:* (undergrad *&* MBA1 samples)				
	1. (A) lose $9 (B) lose $9 after having gained $30 - A hurts more	Cramer’s V	0.37 [0.23, 0.53]	0.50 [0.41, 0.59]	signal-inconsistent, larger
			0.51 [0.39, 0.63]		signal-consistent
	2. (A) lose $9 (B) lose $9 after having lost $30 - B hurts more	Cramer’s V	0.25 [0.10, 0.41]	0.54 [0.45, 0.62]	signal-inconsistent, larger
			0.46 [0.34, 0.58]		signal-consistent
	3. (A) lose $9 (B) lose $9 after having lost $250 - A hurts more	Cramer’s V	*-0.04 [-0.25, -0.00]	0.51 [0.42, 0.59]	signal-inconsistent, opposite
			*-0.30 [-0.42, -0.18]		
	*First group of questions:* (MBA1 sample only)				
	4. (A) lose $9 (B) lose $9 after a loss of $1000 - A hurts more	Cramer’s V	*-0.18 [-0.35, -0.01]	0.47 [0.39, 0.56]	signal-inconsistent, opposite
	5. (A) lose $9 after a loss of $30 (B) lose $9 after a loss of $1000 - A hurts more	Cramer’s V	*-0.14 [-0.31, -0.00]	0.41 [0.33, 0.50]	signal-inconsistent, opposite
	*second group of questions:* (MBA2 sample)				
	1. (A) lose $9 (B) lose $9 after a loss of $9 - B hurts more	Cramer’s V	0.35 [0.20, 0.52]	0.51 [0.43, 0.60]	signal-inconsistent, larger
	2. (A) lose $9 (B) lose $9 after a loss of $18 - B hurts more	Cramer’s V	0.32 [0.18, 0.49]	0.60 [0.51, 0.69]	signal-inconsistent, larger
	3. (A) lose $9 (B) lose $9 after a loss of $36 - B hurts more	Cramer’s V	0.29 [0.14, 0.46]	0.59 [0.50, 0.67]	signal-inconsistent, larger
	4. (A) lose $9. (B) Lose after a loss of $45. - B hurts more	Cramer’s V	0.30 [0.16, 0.47]	0.59 [0.51, 0.68]	signal-inconsistent, larger
	5. (A) lose $9 after a loss of $9. (B) lose $9 after a loss of $36. - B hurts more	Cramer’s V	0.37 [0.22, 0.54]	0.57 [0.48, 0.66]	signal-inconsistent, larger
	**overall**				**mixed: 7 supported, 3 unsupported-opposite**.
7	higher willingness to spend in hotel than in grocery	Cohen’s *d*	—	0.20 [0.03, 0.38]	**successful: signal, supported**
8	friend<stranger	*η²p*	—	0.55 [0.49, 0.59]	signal, supported
	Interaction:friend-closer to cost, stranger-closer to market price	*η²p*	—	0.07 [0.03, 0.12]	signal, supported
	**overall**				**successful, both supported**
9	giving away	Cramer’s V	0.72 [0.58, 0.86]	0.81 [0.72, 0.89]	signal-consistent
	drinking	Cramer’s V	0.80 [0.64, 0.96]	0.77 [0.68, 0.85]	signal-consistent
	giving away versus drinking	Cramer’s V	0.06 [0.00, 0.27]	0.00 [0.00, 0.14]	no signal-consistent
	**overall**				**successful: 3 as expected**
10	investment $400>cost $400	Cohen’s *d* (paired)	~0.50 [~0.29,~0.71]	0.43 [0.33, 0.52]	**successful: signal, supported**
	investment $400>saved $100			0.36 [0.27, 0.45]	
11	prior activity cost - 20 versus 50 (not core)	*η²p*	—	0.02	signal, consistent
	dinner (unrelated) versus sports ticket (related)	Cohen’s *d* (paired)	—	$50: 0.26 [0.13, 0.38] $20: 0.28 [0.15, 0.41]	signal, consistent
	flu vaccination (unrelated) versus sports ticket (related)	Cohen’s *d* (paired)	—	$50: 0.04 [-0.08, 0.17]$20: 0.05 [-0.08, 0.17])	no signal, inconsistent
	**overall**				**mixed: one supported, one unsupported**
12	$15 ticket versus $40 ticket	Cohen’s *d*	0.48 [-0.02, 0.97]	0.23 [0.04, 0.41]	**successful**: signal, inconsistent, weaker
13	won $30: risk-seeking versus risk-averse (loss potential)	Cohen’s *h*	0.41	*-0.46 [-0.54, -0.37]	**unsuccessful:** signal-inconsistent, opposite
14	lost $30: risk-seeking versus risk-averse (loss potential)	Cohen’s *h*	0.20	0.72 [0.63, 0.81]	**successful**: signal-inconsistent, larger
15	lost $30: risk-seeking versus risk-averse (no loss potential)	Cohen’s *h*	0.20	*-0.55 [-0.63, -0.46]	**unsuccessful:** signal-inconsistent, opposite
13−15	Problems 13 and 14 compared (exploratory)	Cohen’s *g*	—	0.20 [0.12, 0.26]	signal; more risk averse following loss
	Problems 13 and 15 compared	Cohen’s *g*	—	0.07 [-0.01, 0.14]	no signal
	Problems 14 and 15 compared (exploratory)	Cohen’s *g*	—	0.12 [0.04, 0.20]	signal, expected direction; gain only^a^
	**overall**				**mixed:** P13/P15 unsuccessful, P14 successful
16	1 bet versus 100 bets	Cohen’s *g*	—	0.36 [0.30, 0.40]	**successful**: signal, supported
17	1 investment versus 25 investments	Cohen’s *g*	—	0.27 [0.20, 0.33]	**successful**: signal, supported
***extensions***					
18	paid $40 versus given by friends	Cohen’s *g*	N/A	0.47 [0.41, 0.49]	signal, supported
19	paid $300, pain, stop preferred over continue playing	Cramer’s V	N/A	0.26 [0.20, 0.33]	signal, inconsistent, opposite
20	paid more ->will wear more	Cohen’s *d*	N/A	0.07 [-0.01, 0.16]	signal, supported
	paid more ->will keep longer	Cohen’s *d*	N/A	0.33 [0.25, 0.42]	no signal, unsupported
	compare: keeping>wearing	Cohen’s *dz*	N/A	0.19 [0.10, 0.28]	signal, supported
21	‘merely 27 cents a day’ more attractive than ‘100 US$ a year’ Independent-samples	Cohen’s *d*	N/A	0.61 [0.39, 0.83]	signal, supported
	dependent-samples	Cohen’s *dz*	N/A	0.29 [0.14, 0.45]	signal, supported

Note. *Original study predicted these would be opposite to the other items. Cramer’s V only includes positive values, yet we converted it in the negative effect when the direction (noted with *) is the opposite.

^a^ We note that Problems 13−15 from Thaler [1, p. 198] were not easy to deduce hypotheses and analyses from. We pre-registered testing each problem and then contrasting 14 and 15 against 13, yet given that Problem 13 was not supported, we realized the need to reframe these contrasts and also run an exploratory contrast between Problems 14 and 15, which in hindsight we should have included a hypothesis for in Stage 1. Overall, we categorized the main demonstration in Problem 13 as failed yet the contrasts to 14 and 15 as aligned with the arguments made, and so as successful. We conclude with our impressions that the demonstrations provided are not empirically aligned with the arguments made, specifically the claim that this is about ‘chance to break even’, and are more likely the result of the contrast between loss potential and no-loss potential. We reframed this post-hoc in Stage 2 throughout to align with that insight.

### 3.1. Replications

#### 3.1.1. Problem 1: Tversky and Kahneman, 1986

In our replication of Problem 1, we found support for Tversky and Kahneman’s [13] findings that people were more risk-averse for gains (84%) than for losses (56%; *χ²*(1) = 85.03, *p* < 0.001; Cramer’s V = 0.41 [0.32, 0.50]).

#### 3.1.2. Problem 2: Tversky and Kahneman, 1981

In our replication of Problem 2, we failed to find support for Tversky and Kahneman’s [6] findings that people were more inclined to spend 20 min to save $5 out of $15 (save $5 out of $15: 11%; save $5 out of $125: 7%; *χ²*(1) = 3.39, *p* = 0.066; Cramer’s V = 0.07 [0.00, 0.17]).

#### 3.1.3. Problem 3: Tversky and Kahneman, 1981

In our replication of Problem 3, we found support for Tversky and Kahneman‘s [6] findings that sunk costs impact decisions less when sunk costs are not from the same mental account (90% bought ticket after losing $10), compared with when sunk costs are from the same mental account (77% bought ticket after losing a ticket; *χ²*(1) = 16.18, *p* < 0.001; Cramer’s V = 0.17 [0.09, 0.27]).

#### 3.1.4. Problem 4: Thaler, 1985

In our replication of Problem 4, we found support for Thaler’s [9] findings that people prefer to segregate gains (Cramer’s V = 0.26 [0.20, 0.33]), integrate losses (Cramer’s V = 0.23 [0.17, 0.29]), integrate smaller losses with larger gains (Cramer’s V = 0.43 [0.37, 0.49]), and segregate small gains from large losses (Cramer’s V = 0.31 [0.25, 0.37]). Effects were consistent with yet weaker than the original studies, and a larger proportion of the participants in the replication were indifferent.

#### 3.1.5. Problem 5: Thaler and Johnson, 1990

In our replication of Problem 5, we found support for Thaler and Johnson’s [8] findings that integrated losses were less upsetting than separate losses (5B: Cramer’s V = 0.28 [0.21, 0.34]; 5C: Cramer’s V = 0.23 [0.17, 0.29]), but opposite findings to the original’s that separate gains were happier than integrated gains (5A: Cramer’s V = 0.09 [0.03, 0.16]).

#### 3.1.6. Problem 6: Thaler and Johnson, 1990

In our replication of Problem 6, we found some support for Thaler and Johnson’s [8] findings: seven effects replicated well with larger effects, whereas three effects were in the opposite direction. Participants perceived the loss of $9 as less upsetting when it occurs after a prior gain (Cramer’s V = 0.50 [0.41, 0.59]), but as more upsetting if the $9 loss followed prior losses, yet—inconsistent with the target article—the magnitude of the prior losses did not seem to have much impact (Cramer’s V = 0.47–0.60). When prior losses were compared directly in the comparison between losing $9 after a loss of either $30 or $1000, participants perceived the loss following $1000 to be more painful, opposite from the target’s findings.

Regardless of the comparison between which hurts more, the core argument is that people are not indifferent to prior losses, and our findings support this idea—participants indicated that they think they would not ignore prior losses and integrate this in their evaluations of loss.

#### 3.1.7. Problem 7: Thaler, 1985

In our replication of Problem 7, we found support for Thaler’s [9] findings that people are willing to adjust their spending based on the purchasing context. People were willing to pay higher prices for the same soda in a fancy resort hotel than in a grocery store (*t*(506) = 2.30, *p* = 0.011; Cohen’s *d* = 0.20 [0.03, 0.38]).

#### 3.1.8. Problem 8: Thaler, 1985

In our replication of Problem 8, we found support for Thaler’s [9] findings that people request a price equal to cost when selling to a friend and a price equal to market price when selling to a stranger (unless their cost exceeds market price) (friend versus stranger: *F* = 594.75, *p* <0.001, *η²p =* 0.55 [0.49, 0.59]; interaction of the three factors—buyer, cost, and market price: *F* = 19.51, *p* < 0.001, *η²p =* 0.07 [0.03, 0.12]).

**Figure 1.**
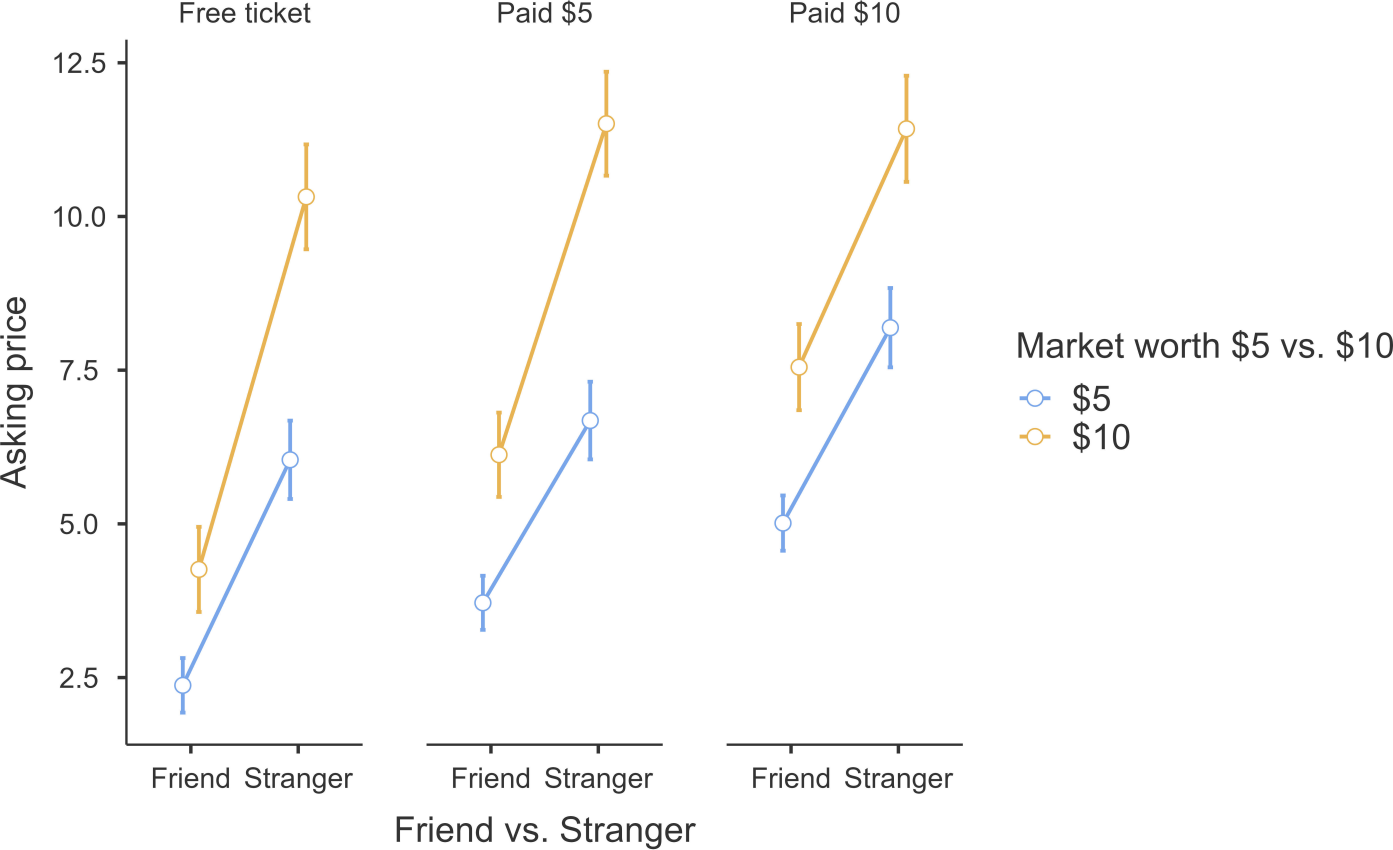
Problem 8: Asking price interaction between buyer closeness, amount paid, and market price.

The interaction pattern generally captured the spirit of having different mental accounts used for determining asking price when selling to friends versus to strangers, yet deviated slightly from the reported findings, as can be seen in figure 1 (plot using JAMOVI ‘jmv’ R package [37]). Amount paid and market price impacted the asking price both for a friend and for a stranger, yet the asking price for a stranger was indeed closer to the market price, whereas the asking price for a friend was closer to cost.

#### 3.1.9. Problem 9: Shafir and Thaler, 1998, unpublished data

In our replication of Problem 9, we found support for Shafir and Thaler’s, 1998, unpublished data, findings that people’s estimation of the cost of a bottle of wine—which gains value over time—differs from a rational economic assessment in which the cost reflects current market price (giving away: *χ*²(4) = 333, *p* < 0.001, Cramer’s V = 0.81 [0.72, 0.89]; drinking: *χ*²(4) = 298, *p* < 0.001, Cramer’s V = 0.77 [0.68, 0.85]; comparing giving away versus drinking: *χ*²(4) = 3.64, *p* = 0.457, Cramer’s V = 0.00 [0.00, 0.14]).

#### 3.1.10. Problem 10: Shafir and Thaler, 1998, unpublished data

In our replication of Problem 10, we found support for Shafir and Thaler’s, 1998, unpublished data, findings that people tend to perceive the wine purchase as an investment (spent $400: *M* = 2.98, s.d.= 1.45; invest $400: *M* = 3.56, s.d. = 1.30; save $100: *M* = 3.08, s.d. = 1.36; *F*(2, 1002) *=* 25.26, *η²p* = 0.05).

#### 3.1.11. Problem 11: Heath and Soll, 1996; Study 2

Problem 11 focused on the mental-budgeting effect. Heath and Soll [3] examined whether people would spend $25 on a theatre ticket, manipulating two factors about events prior to the decision: (i) whether the prior event was an expenditure or a gift, and (ii) whether the event was related (sports ticket) or unrelated (dinner/flu vaccination) to the theatre ticket. They showed that people were less willing to spend money on a theatre ticket the more they previously spent, and especially if it was spent on something that seemed related—such as a sports event ticket, compared with something that was unrelated.

**Figure 2.**
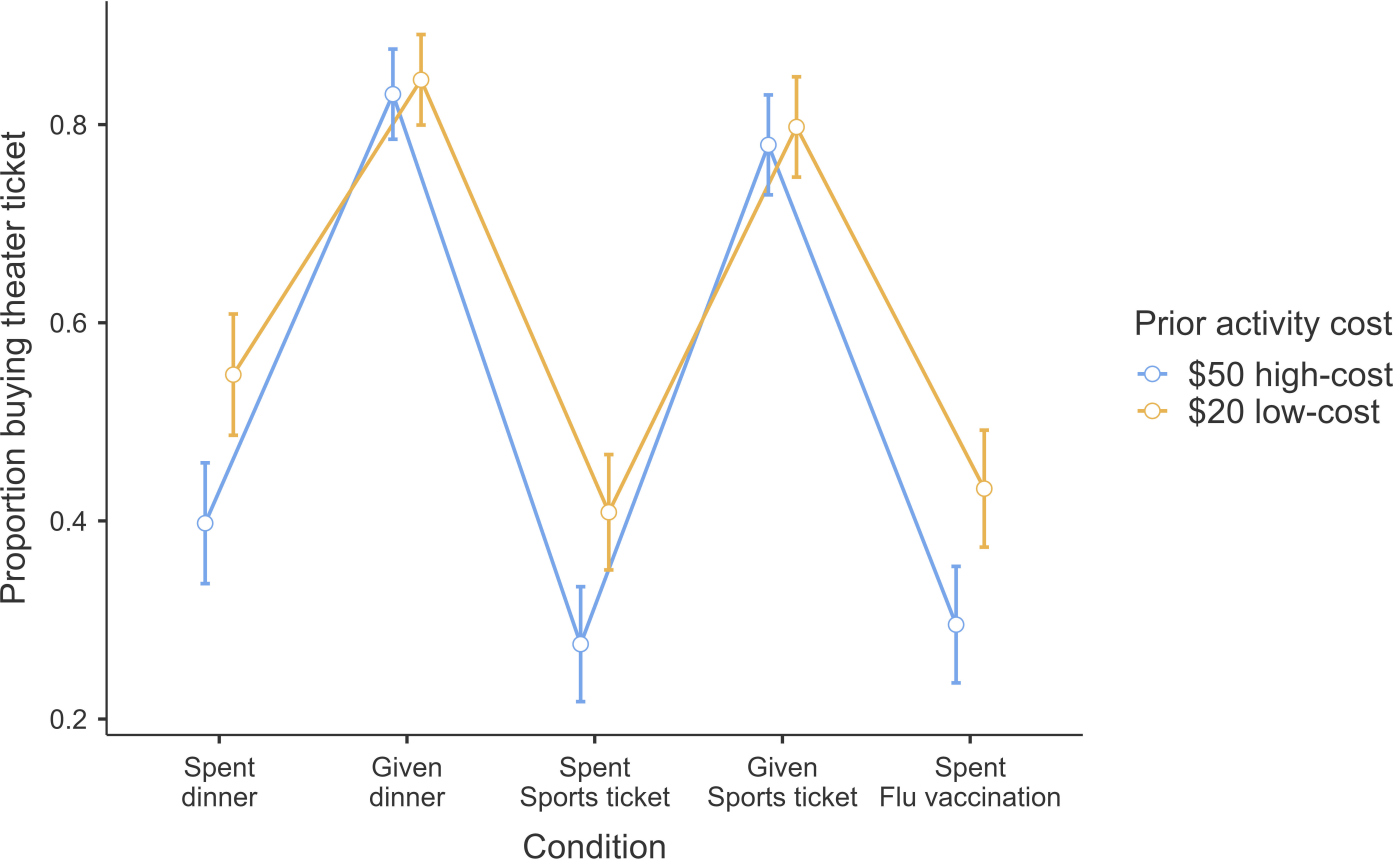
Problem 11: Willingness to buy a theatre ticket after related versus unrelated and given versus spent activities.

In our replication of Problem 11, we found mixed support for their hypothesis. We found the intended pattern for dinner versus sports ticket, and participants were more willing to buy a theatre ticket when they spent their money on the unrelated dinner compared with the related sports event ticket ($50: Cohen’s *d* = 0.26 [0.13, 0.38]; $20: Cohen’s *d* = 0.28 [0.15, 0.41]), yet the findings of the unrelated flu vaccination mirrored that of the related sports event ticket ($50: Cohen’s *d* = 0.04 [−0.08, 0.17]; $20: Cohen’s *d* = 0.05 [−0.08, 0.17]). We plotted the results in figure 2.

#### 3.1.12. Problem 12: Leclerc *et al.*, 1995

In our replication of Problem 12, we found support for Leclerc *et al*.’s [15] findings that the price of a ticket influences people’s willingness to pay for the time to wait to obtain that ticket ($15 ticket: *M* = 8.14, s.d. = 11.32, versus $40 ticket: *M* = 10.34, s.d. = 7.66; Cohen’s *d* = 0.23 [0.04, 0.41]).

#### 3.1.13. Problems 13–15: Thaler, 1999

In Problems 13−15, Thaler [1] manipulated gain and loss scenarios and showed that the outcomes of prior gambles could influence subsequent decisions. In our replication of Problems 13−15, we found mixed evidence for the hypotheses.

We did not find support for prior gains as stimulating risk-seeking behaviour (Problem 13: 28%), yet we found support for a greater inclination towards risk-taking when there is a prior loss (Problem 14: 17% versus Problem 15: 24%). Going beyond the pre-registered singular problem statistical analyses, in Stage 2, we also contrasted each of the three problems and found that prior loss stimulated stronger risk aversion than prior gain (Problem 14 > Problem 13; Cohen’s *g* = 0.20 [0.12, 0.26]), and that in cases of similar prior loss, there was higher risk aversion when there was an additional loss potential (Problem 14 > Problem 15: Cohen’s *g* = 0.12 [0.04, 0.20]; no support for differences between problems 13 and 15: Cohen’s *g* = 0.07 [-0.01, 0.14]).

#### 3.1.14. Problem 16: Samuelson, 1963

In our replication of Problem 16, we found support for Samuelson’s [16] findings that bracketing gambles together increases the attractiveness of individual bets (single coin flip: 25%; 100 coin flips: 49%; Cohen’s *g* = 0.36 [0.30, 0.40]).

#### 3.1.15. Problem 17: Thaler, 1999

In our replication of Problem 17, we found support for Thaler’s [1] finding that narrow framing suppresses risk-seeking behaviours (single project: 30%; 25 investments: 47%; Cohen’s *g* = 0.27 [0.20, 0.33]).

### 3.2. Extensions testing review’s untested predictions

#### 3.2.1. Problem 18: Thaler, 1980

Thaler [12] proposed that families would be more inclined to go to a basketball game in bad weather when they paid for the tickets, compared with when they received the tickets as a gift. In our Problem 18 extension, we found support for this prediction (paid $40: 32%; given by friends: 14%; Cohen’s *g* = 0.47 [0.41, 0.49]).

#### 3.2.2. Problem 19: Thaler, 1980

Thaler [12] suggested that due to the sunk cost effect, people are more inclined to continue playing despite pain if they have already paid a membership fee. However, in our Problem 19 extension, we failed to find support for the prediction, where 76% of the participants chose to stop playing (*χ*² = 132.60, *p* < 0.001, opposite direction to prediction; Cramer’s V = 0.26 [0.20, 0.33]).

#### 3.2.3. Problem 20: Thaler, 1999

Thaler [1] proposed that when people buy a pair of uncomfortable shoes, the more expensive the shoes are, the more times they will be tried on and the longer they will be kept. In our Problem 20 extension, we only found partial support for his prediction. Although our participants were inclined to keep the shoes for a longer time (*t*(506) = 7.53, *p* < 0.001*,* Cohen’s *d* = 0.33 [0.24, 0.42]), they were not as inclined to keep wearing them (*t*(506) = 1.64, *p* = 0.51, Cohen’s *d* = 0.07 [−0.01, 0.16]; Stage 2 added comparison: *t*(506) = 4.31, *p* < 0.001, Cohen’s *dz* = 0.19 [0.10, 0.28])

#### 3.2.4. Problem 21: Thaler, 1999

Thaler [1] predicted that in subscription services people will be more likely to purchase smaller, more often repeating costs over larger, less often costs. In our Problem 21 extension, we indeed found that when presented individually, the framing ‘merely 27 cents a day’ (*M* = 44.53, s.d. = 32.32) was rated as more attractive than ‘100 US$ a year’ (*M* = 26.04, s.d. = 28.05; *t*(331) = 5.57, *p* < 0.001; Cohen’s *d* = 0.61 [0.39, 0.83]). We found similar results when the two options were presented together in a within-subject design (‘merely 27 cents a day’: *M* = 45.16, s.d. = 31.71; ‘100 US$ a year’: *M* = 35.72, s.d. = 29.05; *t*(169) = 3.82, *p* < 0.001; Cohen’s *dz* = 0.29, [0.14, 0.45]).

### 3.3. Comparing replication to original findings

We summarized the findings, the comparison to the original findings, and our interpretation of our findings in comparison to the original findings in table 16.

We used the replication evaluation criteria by LeBel *et al*. [36]. In Stage 1 and our initial submission of Stage 2, we were sometimes unable to deduce the effects. Yet in our revision of Stage 2, we were able to use a guide by Jané *et al.* [23] to provide effect size for most effects in the original studies and for all of the replication effects. In the original problems where we did not have enough information, we simply used ‘signal’ versus ‘no signal’ and the direction of the effect in our replication interpretation.

## 4. Discussion

We carried out a well-powered Registered Report to replicate and extend classic decision-making and behavioural economics problems that were reviewed by Thaler [1] on or related to the topic of mental accounting. More than 26 years after the publication of Thaler’s review paper, in our replication, we were able to find support for 11 out of the 17 problems reviewed. Specifically, we found consistent results for Problems 1, 3, 4, 7–9, 10, 12, 14, 16 and 17. Yet, the results for Problems 5, 6 and 11 were mixed, and the results of Problems 2, 13 and 15 were inconsistent with the original findings. In the following section, we discuss our replication’s findings in comparison with the original studies’, and review the results of the extensions. We then discuss the limitations and promising future directions.

### 4.1. Replications

#### 4.1.1. Problems with mixed results

We categorized our findings regarding Problems 5, 6, and 11 as mixed.

Problems 5 and 6 offered new findings that were not entirely consistent with the original’s findings. In Problem 5, we found no indication of a preference to spread out gains, although if we take a broader view on the comparisons between gains and losses, they seemed to be in the same direction as in the original.

Problem 6 also yielded mixed results. Out of a total of 10 questions, we found support for 7 of them. In agreement with the original findings, people actively integrated the loss of $9 into prior gains but not into prior losses. However, unlike the original, regardless of the magnitude of the previous loss, people seemed more loss averse after the loss.

In Problem 11, the findings followed the expected pattern of higher likelihood to spend on a theatre ticket after spending on an unrelated dinner compared with a related sport event ticket. Yet we did not find an indication for such differences when comparing between the related sport event ticket and the unrelated flu vaccination. This suggests the need for a more comprehensive replication of the studies by Heath and Soll [3] to examine all of their events (e.g. boat tour, party snacks, jeans, watch, etc.) and examine whether the willingness to consume and mentally budget is indeed about relatedness, typicality or perhaps some other factor.

To summarize, this hedonic editing effect was only partially supported under the particular methodology and context. Together with Problem 4, these ambivalent results call for a more precise notion to fully capture the complexity of the hedonic editing effect. Replications, therefore, serve as an important method to identify and set limits on certain effects.

#### 4.1.2. Problems with inconsistent results

Results from Problems 2, 13 and 15 were not in alignment with the original research findings.

Problem 2 examined people’s perceptions of the value of time. The majority of the participants were unwilling to drive 20 min to save $5, regardless of the price. In hindsight, this makes sense and raises an important dilemma in conducting replication studies—whether or not to update and adjust the prices to current days. As one of the participants in the feedback section pointed out, it may cost more than $5 to drive 20 min to the other store with the changing costs of driving and the devaluing of money since the problem was first presented in the 1980s. What would have been plausible to some participants in the 1980s may no longer be plausible to most of current-time participants. Therefore, the inconsistent findings may not be due to lack of an effect, but rather because of the changing circumstances. The differences in the findings could be due to the passage of time and could also be due to our sample’s demographics compared with the original’s, or because of the unified design. The exact reason for the different findings is down to speculation. Future replications could further examine adjustments to the scenarios or to assess different moderating factors.

We felt that it was important to first conduct a direct replication using unadjusted prices because otherwise the differences may have been attributed to our changes. We at least now have the insight that the phenomenon cannot be observed using the same questions in the current context, and future studies can now have a better estimate of the likelihood of replicating the study without adjustments.

Problems 13 and 15 were questions on risk attitudes, with findings that differed from the original claim, with effects in the opposite direction. The prior gain failed to trigger risk-seeking behaviour as anticipated. In fact, the inconsistent result is in congruence with the long-held debate regarding the direction of the impact [38]. According to Merkle *et al*. [38], both risk-seeking and risk-averse behaviours after gains are justifiable. They argued that people can be motivated to be risk-seeking by the house money effect and the hedonic editing hypothesis, or be motivated to avoid risk by the prospect theory. Further research is needed to come up with a more unified explanation for this, and Imas [39] and Merkle *et al.* [38] have already made promising contributions by suggesting the realization effect.

### 4.2. Extensions

Going beyond the replication of original studies that reported empirical evidence, we also ran four extensions to examine the predictions Thaler [1] made or referred to, which were not accompanied by empirical evidence. Among them, we found empirical support for Problems 18 and 21, mixed support for Problem 20 and no support for Problem 19.

Problems 18–20 all targeted the sunk cost effect. In Problem 18, more participants chose to weather the snowstorm and make an effort to go to the sports game when they paid for the ticket as compared with when they received the ticket as a gift. However, in Problem 19, the large majority of the participants said they will not continue playing tennis after suffering an elbow injury, despite the expensive $300 membership fee. In Problem 20, participants agreed that they tend to keep the uncomfortable shoes longer when the price is higher, yet with no indication for the prediction that they would also try to wear them more. Taking into account the replication success of Problem 3, these together revealed that the sunk cost effect might be context-based, such that sunk cost effects may not manifest when there is anticipated physical pain.

We found support for the pennies-a-day effect in Problem 21 [20]. Price frames seemed to affect the comparability of the offers, where expressing the price on a per-day basis helps to lower participants’ price sensitivity [40].

### 4.3. Replications of a review article

This replication project differs from a typical replication registered report, in that rather than focusing on a single empirical study or article, we targeted a review article that covered an entire body of literature on multiple related phenomena with empirical demonstrations from multiple seminal articles. Rather than replicating each independently using different samples, we combined all the studies into a single data collection and mapped out all the effects. In addition to attempting a replication of original studies, we added extensions testing predictions that did not have any reports of findings. Anecdotal evidence and untested assumptions and predictions are useful, as they provide ideas for future research to build on existing empirical studies. Replications and extensions of a review article can help tackle both aspects, by systematically mapping the studies reported as well as untested claims that can be empirically tested. We hope to see more systematic replications and extensions of impactful review papers taking a similar approach to ours.

### 4.4. Limitations and directions for future research

Despite our best efforts to follow the original studies as closely as possible, our replication differed from the originals in several ways, and we had to make many adjustments and analytical decisions. Many of the original studies only reported descriptive statistics, and there were ambiguities regarding the exact analysis used. As a result, we deduced a set of comprehensive statistical analyses that we thought would help better interpret the answers. The lack of analytical details in the original studies raises the importance of reproductions and replications of old studies to facilitate a more transparent sharing of methods, data, code and the documentation needed to facilitate reproducible, replicable future research [41]. We tried our best to compare the original’s findings with the replications’, yet given our reconstruction and adjustments to the data analysis, we caution against over-interpreting the comparisons between the replication results and the original effects.

Our participants were exclusively from the United States and recruited using an online platform, which is a limitation to generalizability [42]. Follow-up studies may aim to rerun the same problems using non-US samples to explore the cross-cultural reliability of the mental accounting phenomenon. For instance, a follow-up mass collaboration project conducted by Priolo *et al*. [19] was a promising attempt in examining the robustness of mental accounting across cultural contexts. In addition, we note that the data collection for this project was conducted during the COVID-19 pandemic. Though we found support for most studies, our participants may show different risk-seeking behaviours compared with non-pandemic periods. For example, Yue *et al*. [43] argued that households altered their risk preference and became more risk-averse due to the pandemic. Thus, the temporal specificity sets another constraint on generalizability.

In this project, we aimed to systematically revisit experiments testing different accounts of the mental accounting framework reviewed by Thaler [1]. We focused on the empirical aspects of the singular problems and did not go further to try and discuss implications for mental accounting theory as a whole. Therefore, the results of our replications for each of the problems should be interpreted separately and cautiously. We also did not address the mental accounting tendency at the individual level. Following the suggestion from one of the reviewers, we encourage future research to delve deeper in this regard, to undertake broader theoretical integrations and examine individual tendencies. We see much promise in further studies of the links between the different aspects of the mental accounting framework.

## 5. Conclusion

We examined the replicability of the mental accounting studies summarized by Thaler [1]. We successfully replicated 11 problems, found mixed support for three problems and failed to find support for three problems, suggesting that mental accounting effects generally tend to replicate well but that some effects may be more complex and contextual than originally documented. We believe our reconstruction and reanalysis of classic experiments, as well as our exploratory analyses, provide an impetus and practical guide to stimulate further follow-up research to examine the mental accounting phenomenon as a whole.

## Data Availability

We provided all materials, data, and code on the Open Science Framework (OSF) [44]. Supplementary material is available online [45].
